# Case Report: Lung Adenocarcinoma Initially Presenting With Cutaneous and Subcutaneous Metastases

**DOI:** 10.3389/fonc.2022.925382

**Published:** 2022-07-12

**Authors:** Jingjing Wang, Ruolin Wu, Fang Liu, Liu Yang, Fan Hu, Zhijian Wu, Zairong Gao, Xiaotian Xia

**Affiliations:** ^1^Department of Nuclear Medicine, Union Hospital, Tongji Medical College, Huazhong University of Science and Technology, Wuhan, China; ^2^Hubei Province Key Laboratory of Molecular Imaging, Wuhan, China; ^3^Key Laboratory of Biological Targeted Therapy, the Ministry of Education, Wuhan, China; ^4^Department of Dermatology, Union Hospital, Tongji Medical College, Huazhong University of Science and Technology, Wuhan, China; ^5^Department of Nuclear Medicine, The People’s Hospital of Honghu, Honghu, China

**Keywords:** lung adenocarcinoma, soft tissue, skin rashes, metastasis, ^18^F-FDG, PET/CT

## Abstract

Cutaneous and subcutaneous soft tissue metastases are rare in lung adenocarcinoma and suggest poor prognosis. We report a patient with lung adenocarcinoma who initially presented with cutaneous and subcutaneous metastases to the abdomen that were initially presumed to be herpes zoster and an occult subcutaneous soft tissue mass. Because the lesions progressed over 3 weeks despite routine herpes zoster treatment, magnetic resonance imaging was performed and showed a presumed sarcoma; however, ^18^F-fluourodeoxyglucose positron emission tomography/computed tomography demonstrated pulmonary lesions. Biopsy of the abdominal lesion confirmed poorly differentiated lung adenocarcinoma. Early diagnosis of soft tissue metastasis can be difficult. Clinicians should suspect internal organ malignancy when a progressive cutaneous or subcutaneous soft tissue lesion is encountered.

## Introduction

Lung cancer is a frequently encountered malignancy that can metastasize to almost all organs and is associated with high mortality (1, 2). Lung adenocarcinoma commonly metastasizes to the liver, adrenal glands, brain, and bone (3). Soft tissue metastases from lung adenocarcinoma are rare and occur predominantly in men (4). They may be apparent before the primary tumor and typically herald a poor prognosis. Reported mean survival in patients with skin metastases is 2.9 months (5), so early diagnosis and treatment are important. However, the diagnosis of skin metastases may be delayed or missed. A high index of suspicion is required.

## Case Description

**Figure 1:**
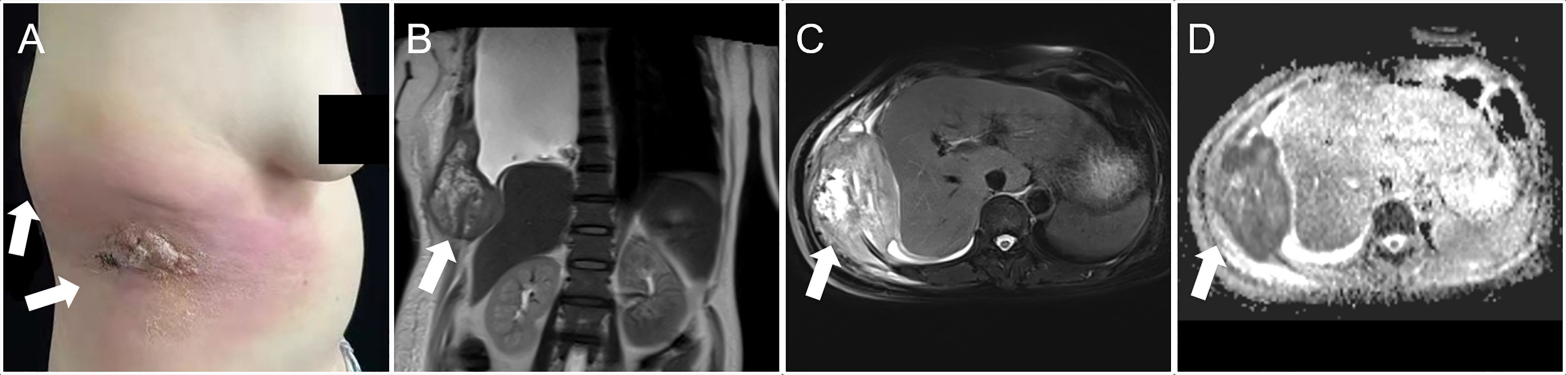
Physical examination showed an erythematous rash and swelling surrounding a skin mass on the right abdomen (**A**, arrows). Magnetic resonance imaging shoed an inhomogeneous soft tissue mass approximately 10 cm in diameter (**B**, coronal T2-weighted image; **C**, axial fat saturation T2-weighted image). The lesion also showed markedly restricted diffusion on diffusion-weighted sequences (**D**, arrow).

**Figure 2:**
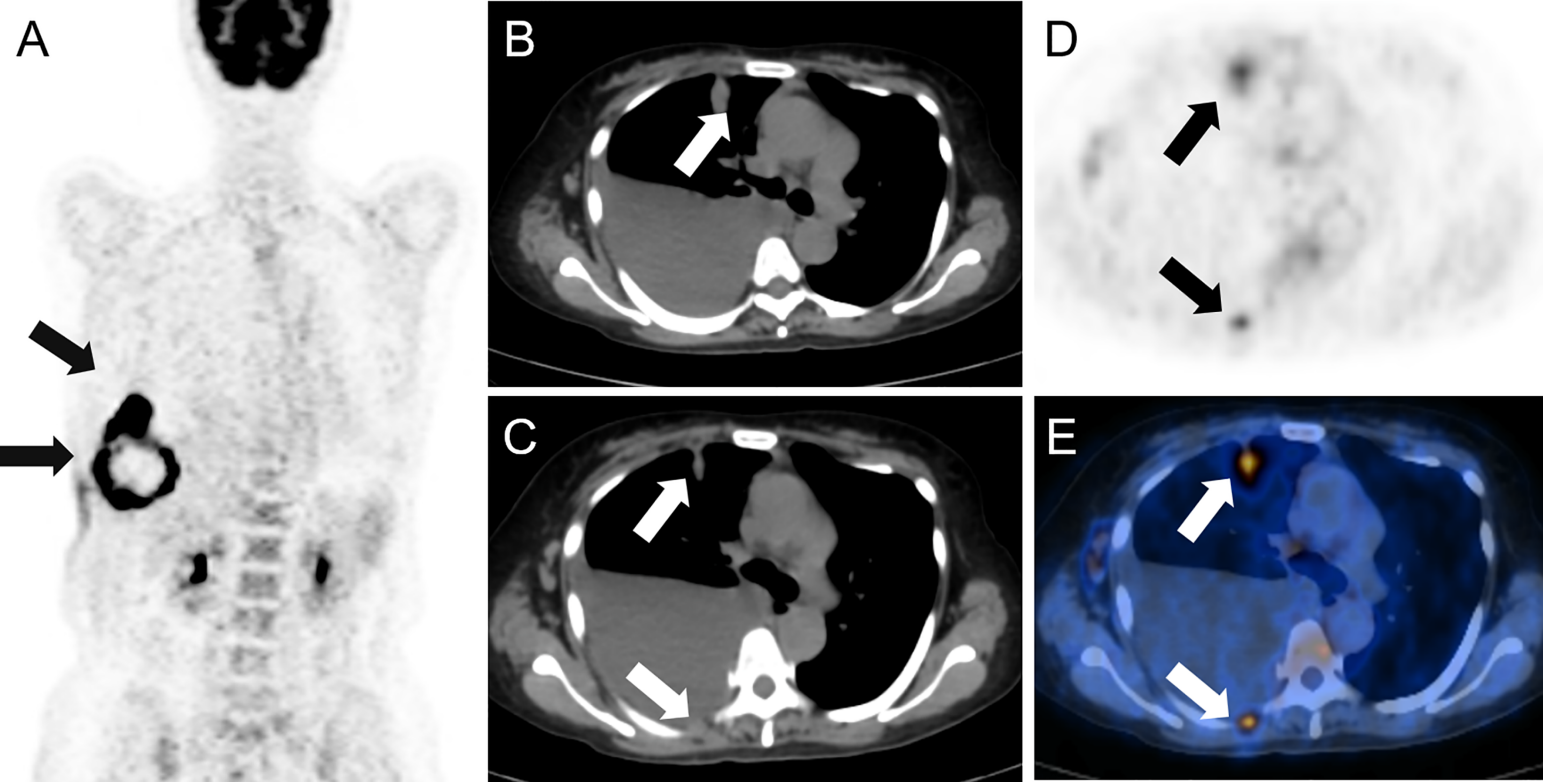
** **A large mass showing hypermetabolism peripherally and hypometabolism centrally was found on 18F-fluorodeoxyglucose positron emission tomography/computed tomography **(A)**, arrows. Hypermetabolic lesions were imaged in the right lung and the posterior pleural wall (arrows) on axial computed tomography **(B, C)**, positron emission tomography **(D)** and fusion imaging **(E)**.

**Figure 3:**
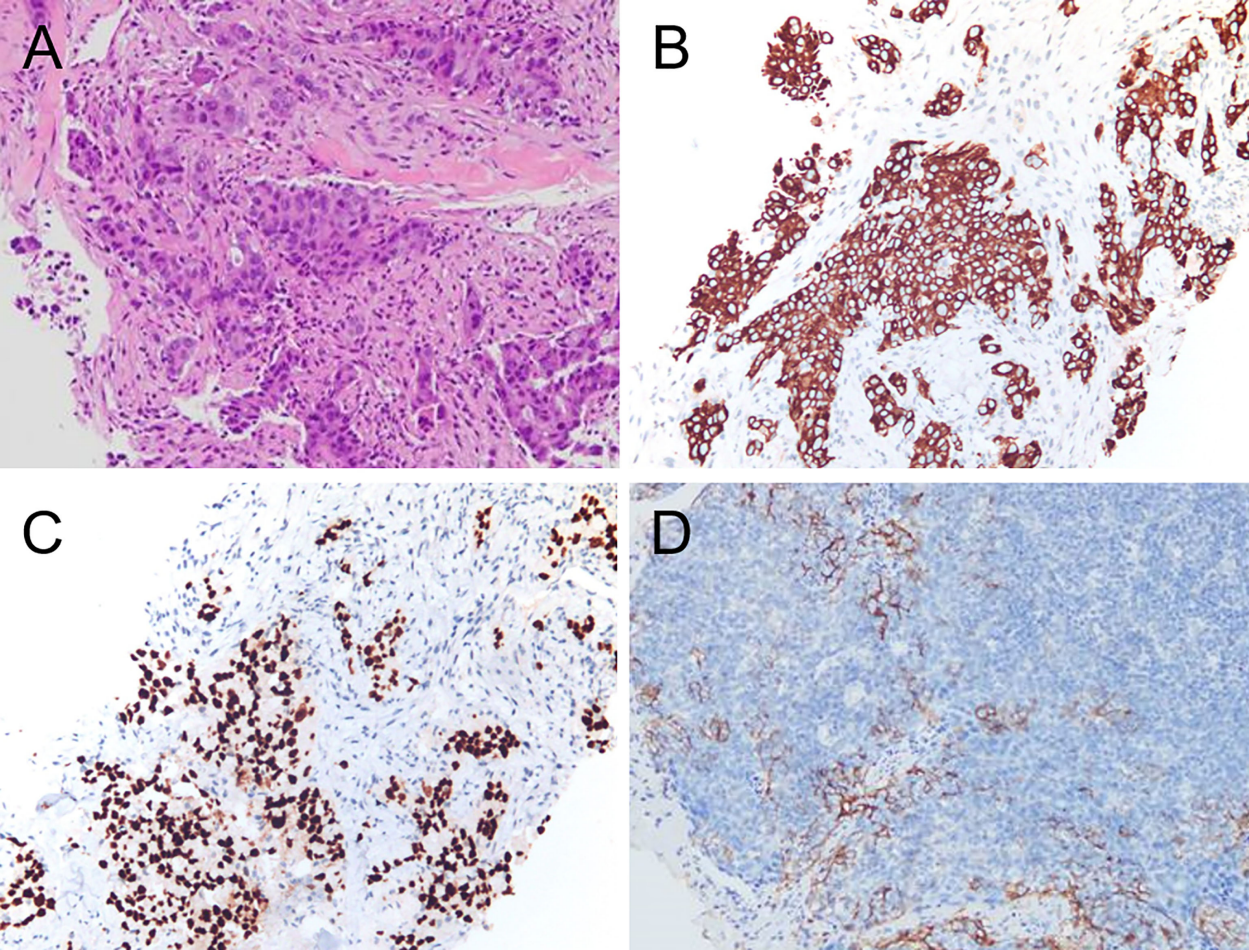
Hematoxylin and eosin staining revealed the tumor was composed of abundant oval and plump cells with enlarged nuclei and red, broad cytoplasm **(A)**. Immunohistochemical staining for CK 7 **(B)** showed a strong and diffuse brown cytoplasmic reaction. TTF-1 staining **(C)** Showed strong nuclear staining of tumor cells. Immunohistochemical analysis showed PDL-1 expression **(D)**.

A 52-year-old woman presented with a 3-week history of painful rash and subcutaneous soft tissue mass overlying the right abdomen at the waistline. She denied constitutional symptoms such as fever, chills, night sweats, and unintentional weight loss. There was no history of major trauma, surgery, smoking, alcohol use, or drug or food allergy. Notably, the patient was exposed to secondhand smoke from nicotine cigarettes due to her husband’s smoking. In addition, her father died of esophageal cancer. Herpes zoster was initially suspected but appropriate treatment did not result in clinical improvement. In fact, progression had occurred. Therefore, she was hospitalized for further investigation and treatment. Physical examination showed a raised skin mass surrounded by swelling and erythema on the right abdomen (**Figure 1A**). Serum erythrocyte sedimentation rate, C-reactive protein, white blood cell count, and multiple tumor markers were elevated. Ultrasonography revealed a solid mass underneath the rash. On magnetic resonance imaging (MRI), the mass was 10 cm in diameter and inhomogeneous on T2-weighted sequences (**Figures 1B, C**) and exhibited markedly restricted diffusion on diffusion-weighted sequences (**Figure 1D**). The mass was suspected to be a sarcoma. To investigate potential distant metastases, ^18^F-fluourodeoxyglucose (FDG) positron emission tomography (PET)/computed tomography (CT) was performed, which showed the previously demonstrated large abdominal mass was hypermetabolic in the periphery and hypometabolic in the center (**Figure 2A**); other hypermetabolic lesions were shown in the right lung and the posterior pleural wall (**Figure 2B–E**). Lung cancer with metastases was suspected and the patient underwent ultrasound-guided biopsy of the subcutaneous soft tissue mass. Examination of hematoxylin and eosin-stained specimen (**Figure 3A**) revealed abundant oval and plump cells with enlarged nuclei and red, broad cytoplasm. Immunohistochemical examination showed staining was positive for CK7 (**Figure 3B**), TTF-1 (**Figure 3C**), and PCK but negative for P63, CK20, Villin, ER, CDX2, HER2, P16, GATA-3, and VT-1. This suggested a diagnosis of primary pulmonary adenocarcinoma with metastasis. Because PDL-1 was expressed (**Figure 3D**) and EGFR mutation was not detected, the patient was placed on bevacizumab plus pemetrexed–platinum doublet chemotherapy. After six cycles, the primary pulmonary lesions shrunk but the cutaneous lesions did not. Molecular testing revealed mutation in the BRAF 15 exon and targeted therapy was proposed, but the patient refused for financial reasons. For relieving the patient’s pain, palliative radiotherapy was initiated.

## Discussion

Lung cancer morbidity and mortality is highest of all cancers (1, 2) and lung adenocarcinoma accounts for approximately 40% of all lung cancers (6). Although lung carcinoma can metastasize to all organs, the liver, adrenal glands, bone, kidney, and brain are the most common sites (3). Metastasis to cutaneous and subcutaneous soft tissues is rare, with reported incidence rates ranging between 1% and 12% (5, 7–9). Soft tissue metastasis can be challenging to diagnose when it is the initial cancer manifestation, as in our patient, who presented with a painful rash in the absence of typical lung adenocarcinoma symptoms (10). Soft tissue metastases may rapidly progress when the initial diagnosis is missed.

To evaluate soft tissue metastases, MRI is the most sensitive and specific imaging modality and enables assessment of tissue characteristics, tumor extent, and areas of reactivity (11, 12). In our patient, MRI was highly suspicious for sarcoma but ^18^F-FDG PET/CT suggested a lung primary, which was confirmed by biopsy. Although MRI can distinguish between benign and malignant tumors, it cannot further distinguish malignancy. Compared with sarcoma, soft tissue metastases from organ malignancies are rare. They are easily missed, especially when symptoms of the primary are absent or atypical. Therefore, ^18^F-FDG PET/CT before biopsy is essential to improve diagnostic accuracy and distinguish soft tissue masses.

Optimal management requires accurate diagnosis, which requires biopsy in most cases (13, 14). In our patient, histopathological and immunohistochemical examinations resulted in a diagnosis of poorly differentiated pulmonary adenocarcinoma (15, 16). In this disease, the appearance of metastatic soft tissue masses indicates an advanced stage and poor prognosis. Chemotherapy, immunotherapy, targeted therapy, and radiotherapy are the mainstay treatments for soft tissue metastasis; surgery is not typically recommended (17–19). Unfortunately, six cycles of bevacizumab plus pemetrexed–platinum doublet chemotherapy were not as effective as we had hoped. The targeted therapy has been shown to decrease tumor burden, decrease symptoms, and dramatically improve survival outcomes in advanced lung cancers (19, 20). However, our patient refused the targeted therapy for financial reasons. Then palliative radiotherapy was initiated and proved effective for pain relief. To date, the patient’s general condition has remained stable.

Early diagnosis of soft tissue metastasis can be difficult. Clinicians should suspect internal organ malignancy when a progressive cutaneous or subcutaneous soft tissue lesion is encountered. A thorough examination should be performed and ^18^F-FDG PET/CT should be considered for further evaluation.

## Data Availability Statement

The raw data supporting the conclusions of this article will be made available by the authors, without undue reservation.

## Ethics Statement

The studies involving human participants were reviewed and approved by Ethical Committee of Union Hospital, Tongji Medical College. The patients/participants provided their written informed consent to participate in this study.

## Author Contributions

JW, RW, LY, ZW and XX obtained and analyzed the clinical data. JW and XX wrote the manuscript. FL and FH designed and constructed the figures. XX and ZG designed the study. All authors contributed to patient care and writing and revising the manuscript and figures. All authors contributed to the article and approved the submitted version.

## Funding

This research was supported by the National Natural Science Foundation of China (Grant Numbers 81801737, 81771866).

## Conflict of Interest

The authors declare that the research was conducted in the absence of any commercial or financial relationships that could be construed as a potential conflict of interest.

## Publisher’s Note

All claims expressed in this article are solely those of the authors and do not necessarily represent those of their affiliated organizations, or those of the publisher, the editors and the reviewers. Any product that may be evaluated in this article, or claim that may be made by its manufacturer, is not guaranteed or endorsed by the publisher.
